# Editorial: Impact of hyperglycemia induced oxidative stress in genetics and epigenetics of metabolic diseases

**DOI:** 10.3389/fgene.2023.1123665

**Published:** 2023-01-26

**Authors:** Saheem Ahmad, Firoz Akhter, Khurshid Ahmad, Saif Khan

**Affiliations:** ^1^ Department of Medical Laboratory Sciences, College of Applied Medical Science, University of Hail, Hail, Saudi Arabia; ^2^ Department of Biomedical Engineering, Stony Brook University, New York, NY, United States; ^3^ Department of Medical Biotechnology, Yeungnam University, Gyeongsan, Republic of Korea; ^4^ Department of Basic Medical and Dental Sciences, College of Dentistry, University of Hail, Hail, Saudi Arabia

**Keywords:** hyperglycemia, metabolic disorders, oxidative stress, genetics, epigenetics

This Research Topic brings together various contributions that highlight cutting-edge methods and analyses for dealing with oxidative stress brought on by hyperglycemia in data on the genetics and epigenetics of metabolic diseases that have been collected using a variety of modalities, both real and simulated. With an emphasis on the connection between hyperglycemia, oxidative stress, genetics, and epigenetics, these methods help us understand the mechanisms behind metabolic disorders. The oxidative stress and hyperglycemia result in the non-enzymatic glycation reaction, which is one of the primary causes of metabolic disorders like diabetes, cancer, *etc.* (Akhter et al., 2014; Akhter et al., 2017; Ahmad et al., 2018a; Shahab et al., 2018).

There are numerous studies that hold the role of hyperglycemia and glycation reaction in cancer state disease (Ashraf et al., 2016; Ahmad et al., 2018b; Ahmad et al., 2018c; Jabir et al., 2018; Khan et al., 2018). Our research team has recently demonstrated the significance of the non-enzymatic glycation process in the epigenetics of cancer (Rehman et al., 2022).

The first article on this subject (Goel et al.), introduces metformin and multiple mechanisms (excluding AMPK activation) through which Metformin may have positive effects in these environments and includes Embase and PubMed/MEDLINE searched evidence that metformin has diversified effects on the systems of the human body. It has been demonstrated to have antioxidant, anti-inflammatory, metabolic, cardioprotective, neuroprotective, antibacterial, and anti-cancer actions. Recently, it has also been discovered to have efficacy in contrast to SARS-CoV-2. The AMPK pathway was identified as one among them all for its effectiveness and efficiency. The authors demonstrate its grand potential, demonstrating that it is capable of being included in treatment plans for conditions other than type 2 diabetes mellitus (T2DM).

Using a different strategy, Bai et al. investigate the relationship between autophagy-related biomarkers in Diabetic Nephropathy by examining the differentially expressed genes associated with autophagy (DEARGs) between Diabetic Nephropathy and samples of healthy renal tubules. They identified a new biomarker of autophagy linked to tubulointerstitial damage in DN (Bai et al.). The author uses bioinformatics analysis to verify the 10 DN patients’ renal tubules’ gene expression profiles, 24 healthy controls, and 43 DEARGs. Protein-protein interactions were used to screen for the hub gene prolyl 4-hydroxylase subunit beta (P4HB), which was then confirmed by using additional datasets and stimulating HK-2 cells under conditions of high glucose concentration. On DEARGs, association analysis, Gene Ontology (GO), and Kyoto Encyclopedia of Genes and Genomes (KEGG) searches were made. Their findings showed a correlation between renal function and P4HB expression in renal tubules. Additionally, a methodology of this research that forecasts data from the GEO database on the gene expression of human renal tubulointerstitial was also demonstrated (GSE30122). In summary, their research revealed P4HB as a new DN-related autophagy biomarker and offered fresh perspectives for understanding the molecular mechanisms underlying Diabetic Nephropathy.

Xi, Zhang, et al., 2022, proposed a unique strategy, where ribose and glucose were glycated *in vitro* to confirm their impact upon epigenetics such as histone methylation and demethylation Xi et al. Specifically, Xi et al. report that after treatment with D-ribose glycated BSA, in SH-SY5Y cells, demethylation of histone 3 (H3K4) was seen together with noticeably elevated amounts of plant homeodomain finger protein eight and lysine-specific demethylase-1 (LSD1) (PHF8). Furthermore, the scientists verified that the presence of D-ribose glycated protein caused considerable demethylation of H3K4me3, H3K4, and H3K4me2. Further evidence of histone demethylation was found in the cell-culture media of SH-SY5Y cells along with D-ribose glycated BSA, where formaldehyde levels had increased. This study offers a fresh understanding of the ribose metabolic disorders-related epigenetic mechanism of diabetic Mellitus (DM). One publication has been contributed to this Research Topic regarding the development of autoantibodies against reactive oxygen species (ROS) modified Histone H1 Protein and its connection to lymphoma.

The design of new technical tools for structural and functional analysis can also benefit from information-based measures. Wang et al. investigated the relationship between the information content of oxidative stress and varicocele. Clinical palpation reveals varicocele as the main factor impacting male fertility. Although the majority of juvenile varicocele cases are asymptomatic, as patients age and the varicocele’s course worsen, symptoms such as testicular atrophy, infertility, and scrotal pain and discomfort gradually develop. These symptoms have an enormous effect on a patient’s physical and mental health. In the setting of a drop in fertility, the author described damage from oxidative stress’s significance in the molecular biology and pathophysiology of varicocele.

In the Binsaleh et al. study (Binsaleh et al.), the histone H1 (His-h1) protein was altered by ROS. Evaluations of the chemical and structural changes were made. To comprehend ROS and alter histone H1, individuals with Hodgkin lymphoma and non-Hodgkin lymphoma were chosen. Then, using biochemical and immunological methods, serum autoantibodies, inflammatory cytokines, and indicators of oxidative stress were investigated. The Langmuir plot was also used to evaluate the development of immunological complexes between antigens and antibodies. The findings imply that the protein’s cryptic neo-epitopes, which autoantibodies were produced against, are exposed by the ROS alterations of histone H1. These alterations may have an impact on how histone DNA interacts, and they may also be associated with gene dysregulation. Their findings indicate that these minor molecular changes coupled with immunological dysregulation may worsen the disease. Developing a new technical instrument for structural and functional analysis can also benefit from information-based measures. We hope that this Research Topic will serve as a helpful resource for the reader regarding the most recent developments in the rapidly expanding field of tools based on information theory and utilized in the study of neuroscience.
